# Substantial fibrin amyloidogenesis in type 2 diabetes assessed using amyloid-selective fluorescent stains

**DOI:** 10.1186/s12933-017-0624-5

**Published:** 2017-11-02

**Authors:** Etheresia Pretorius, Martin J. Page, Lize Engelbrecht, Graham C. Ellis, Douglas B. Kell

**Affiliations:** 1 Department of Physiological Sciences, Faculty of Health Sciences, Stellenbosch University, Stellenbosch Private Bag X1 Matieland, Stellenbosh, 7602 South Africa; 2 Central Analytical Facility, Stellenbosch University, Stellenbosch Private Bag X1 Matieland, Stellenbosh, 7602 South Africa; 3 Synexus Helderberg Clinical Research Centre, Helderberg Synexus South Africa, 7G&H Arun Place, Somerset West, 7130 South Africa; 4 School of Chemistry and Manchester Institute of Biotechnology, The University of Manchester, 131 Princess St, Manchester, M1 7DN Lancs UK

## Abstract

**Background:**

We have previously shown that many chronic, inflammatory diseases are accompanied, and possibly partly caused or exacerbated, by various coagulopathies, manifested as anomalous clots in the form of ‘dense matted deposits’. More recently, we have shown that these clots can be amyloid in nature, and that the plasma of healthy controls can be induced to form such clots by the addition of tiny amounts of bacterial lipopolysaccharide or lipoteichoic acid. Type 2 diabetes (T2D) is also accompanied by raised levels of LPS.

**Methods:**

We use superresolution and confocal microscopies to investigate the amyloid nature of clots from healthy and T2D individuals.

**Results:**

We show here, with the established stain thioflavin T and the novel stains Amytracker**™** 480 and 680, that the clotting of plasma from type 2 diabetics is also amyloid in nature, and that this may be prevented by the addition of suitable concentrations of LPS-binding protein.

**Conclusion:**

This implies strongly that there is indeed a microbial component to the development of type 2 diabetes, and suggests that LBP might be used as treatment for it and its sequelae.

## Introduction

Inflammation is characterised by dysregulated circulating pro-inflammatory molecules, and such molecules have a pathological effect on the haematological system. This is true not only of the immune cells, but also of erythrocytes (RBCs), platelets and plasma proteins such as fibrin(ogen). During inflammation, RBCs experience structural and biochemical changes, which include membrane changes that may be visible as agglutination, eryptosis or microparticle formation [1–7]. In addition, the structure of the fibrin(ogen) protein changes, and this results in anomalous clot formation when fibrinogen is hydrolysed by thrombin [8–11]. Haematological pathology therefore both reflects and is reflected by a pro-coagulant state, which is a hallmark both of inflammation and of the concomitant dysregulated profile of circulating pro-inflammatory molecules [8].

Type 2 diabetes (T2D) is an inflammatory condition that is also characterised by various dysregulated cytokines and other molecules that are immunomodulatory and of pathophysiological importance [12, 13]. This condition is accompanied by many cardiovascular complications, including a thrombotic propensity; however, the inflammatory stimulus is often unknown. Importantly, T2D plasma has aberrant fibrin(ogen) packaging, and this is associated with amyloid fibril formation, as demonstrated by the amyloid-selective stain thioflavin T (ThT) (see [14, 15]) that binds to anomalous clots prepared by adding thrombin to diabetic plasma.

We have recently highlighted the possible and potent role of the circulating bacterial-derived inflammagens lipopolysaccharide (LPS) and lipoteichoic acids (LTAs) on anomalous blood hypercoagulation. LPSs derive from the membranes of Gram-negative bacteria [16], while LTAs originate from membranes of Gram-positives [17]. Importantly, the literature does support the increased circulating LPS in T2D [18–25].

LPS and LTA added to healthy plasma cause amyloidogenic changes in fibrin(ogen), demonstrated by thioflavin T (ThT) binding as well as binding of luminescent conjugated oligothiophene dyes (LCOs), marketed under the trade name Amytracker™, that also stain classical amyloid structures [11]. Various fluorescent markers have been shown to illuminate amyloids (e.g. [26–42]). This includes the luminescent LCO markers commercialised as Amytracker™ 480 and 680 (based on the published molecules HS163 and HS169 [43–45]), developed by Nilsson and colleagues [43, 44, 46–52].

**Fig. 1:**
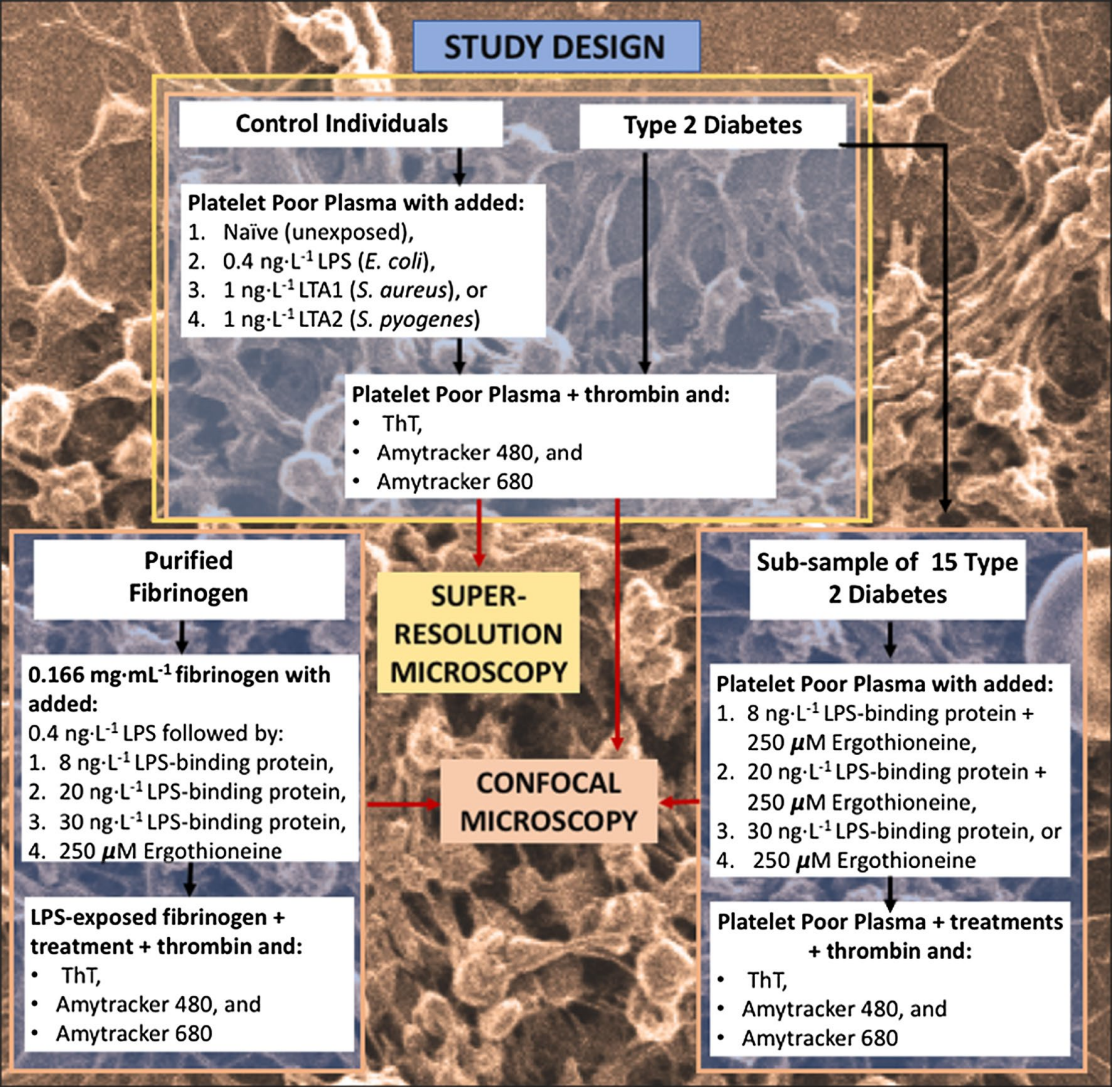
Infographic of workflow and study design

Here we use these stains to provide evidence for a link between T2D as an inflammatory disease and the presence of amyloid proteins. We also assess the effect of different concentrations of LPS-binding protein and the antioxidant l-(+)ergothioneine (see, e.g. [53, 54]) on T2D plasma, and on purified fibrinogen after addition of LPS, to determine if these can decrease amyloid formation and the extent to which this could be shown with the two LCO dyes and ThT (see Fig. 1; infographic of workflow).

## Materials and methods

### Sample population

The control sample consisted of 17 age- and gender-matched (to the T2D sample) healthy individuals and an additional 17 young healthy individuals. Inflammation is known to increase with age [55], so we first sought to determine if there were differences between the two control groups (young versus old), to define if age plays a role in our experimental design. This said, it is well-known that the haematological system is only modestly affected by ageing [56]. Exclusion criteria for the healthy samples were: known (chronic and acute) inflammatory conditions such as asthma, human immunodeficiency virus (HIV) or tuberculosis; auto-immune conditions; risk factors associated with metabolic syndrome; smoking; and, if female, being on contraceptive or hormone replacement treatment. This population did not take any anti-inflammatory medication. Based on these exclusion criteria we classified these control donors as ostensibly healthy. We included individuals in our control group that had a range of BMI values, and for some purposes we grouped them as individuals with a BMI of either < 25 (normal BMI) or ≥ 25 (overweight). The distribution of normal versus overweight healthy individuals were: age controlled healthy individuals: < 24.9: 47%; > 25: 53% and for the younger healthy individuals: < 24.9: 88%; > 25: 12%. Although we included overweight, seemingly healthy individuals in our control group, we recognise that it is well-known that several key inflammatory markers have been consistently associated with obesity, which suggests that a persistent, low-grade, inflammatory response is a potentially modifiable risk factor [57]. Therefore, we acknowledge that overweight apparently healthy individuals may be more prone to low-grade systemic inflammation and that this may impact on their coagulation health.

Our T2D sample consisted of 33 individuals with BMI > 25 (overweight). T2D individuals were voluntarily recruited from a Diabetic Clinic in Somerset West, South Africa. Auto-immune diseases were set as exclusion criteria. Diabetic individuals were diagnosed by an Endocrinologist per the Society for Endocrinology, Metabolism and Diabetes of South Africa Type 2 Diabetes Committee (SEMSDA) guidelines [58]. These guidelines follow the American Diabetes Association (ADA) criteria to define type 2 diabetes. Demographic data including age, gender, as well as clinical information including haemoglobin A1c (HbA1c) levels and medication used by the T2D patients were obtained. Inclusion criteria consisted of both male and female participants, aged 19 and older, with a diagnosis of T2D for more than 3 months prior to screening and without any signs of infection. Smoking and either contraceptive or hormone replacement treatment were exclusion criteria. Whole blood (WB) of all the participants was obtained in citrate tubes. A platelet poor plasma (PPP) isolate was prepared by centrifuging WB for 15 min at 3000*g*.

### Fluorescent markers and inflammagen binding agents

We aimed to determine if the hypercoagulable clot structure that we have previously noted in T2D and confirmed by staining T2D PPP with thioflavin T (ThT), was indeed amyloid in nature [59]. We have also previously shown that both the LCO dyes Amytracker™ 480 and 680 bind to amyloid areas in clots of healthy PPP exposed to known fibrin–amyloidogenic molecules (viz., iron, lipopolysaccharide (LPS) from gram negative bacteria, and two lipoteichoic acids (LTA1 from *Staphylococcus aureus* (Sigma, L2515) and LTA2 from *Streptococcus pyogenes* [Sigma, L3140)] [11]). It was thus of interest to assess if these amyloid markers and ThT will bind to amyloid proteins in clots of T2D in a similar pattern as in the healthy PPP incubated with amyloidogenic molecules that induced amyloidogenic areas (as previously shown in [11]). In addition, we investigated if we could induce a lowered LCO binding in T2D plasma after the addition of LPS-binding protein (LBP) (Abcam, AB119721) and/or an antioxidant compound, ergothioneine (Sigma E7521), to the PPP of T2D (previously we confirmed a reduced binding of ThT in T2D plasma after the addition of LBP [10]). Here, we analysed all diabetes versus controls, but because of the expense and time it takes for the analysis on the confocal microscope, we only looked at a random number of 15 diabetes patents that we additionally exposed to LBP and/or Ergothioneine. We also added LPS followed by LBP to purified fibrinogen to confirm this principle that LPS presence in T2D blood is one of (the main) causes of an amyloidogenic fibrin(ogen) structure (see methods in next section).

The LPS used was from *E. coli* O111:B4 (Sigma, L2630). A final LPS exposure concentration to plasma and purified fibrinogen of 0.4 ng L^−1^, and final LPS-binding protein (LBP) exposure concentrations of 8, 20 and 30 ng L^−1^ were used. A final exposure concentration of 250 µM ergothioneine (Sigma, E7521) was used. Purified fibrinogen (Sigma, F3879) was made up to 0.166 mg mL^−1^.

### Confocal and super-resolution structured illumination (SR-SIM) microscopy of platelet poor plasma (PPP) clots

Platelet poor plasma (PPP) of all healthy and T2D individuals were prepared (as mentioned above), followed by storage at − 80 °C. On the day of analysis, the − 80 °C-stored PPPs were brought to room temperature, followed by a 30-min incubation with ThT at a final concentration of 5 µM and Amytracker™ 480 and 680 (0.1 µL into 100 µL PPP). LBP (8, 20 and 30 ng L^−1^ final exposure concentration), and ergothioneine (250 µM, final exposure concentration) were also added before addition of fluorescent markers. LBP and/or ergothioneine incubation time was 1 h. Before viewing clots on the confocal microscope, thrombin was added in the ratio 1:2, (5 µL thrombin: 10 µL) to create extensive fibrin networks. One clot was prepared for each individual and different random areas were imaged multiple times. Thrombin was provided by the South African National Blood Service, and the thrombin solution was at a concentration of 20 U mL^−1^ and made up in PBS containing 0.2% human serum albumin (see https://www.sigmaaldrich.com/content/dam/sigma-aldrich/docs/Sigma/Product_Information_Sheet/1/t6884pis.pdf for a description of how thrombin units are calculated). A coverslip was placed over the prepared clot, and samples were viewed using a Zeiss LSM 780 with ELYRA PS1 confocal microscope with a Plan-Apochromat 63x/1.4 Oil DIC objective. The following settings were used:

- For ThT: the 488 nm excitation laser was used, with emission measured at 508–570 nm;
- For Amytracker™ 480: the 405 nm excitation laser was used, with emission measured at 478–539 nm; and,
- For Amytracker™ 680: the 561 nm excitation laser was used, with emission measured at 597–695 nm.

A selection of micrographs of the prepared clots were captured. Gain settings were kept the same during all data capture and used for statistical analyses; however, brightness and contrast were slightly adjusted for figure preparation. We captured the fluorescent signal of each of the three fluorescent markers as a composite.czi file in the Zeiss ZEN software and then used ImageJ (FIJI) to split and analyse the RGB channels.

### Quantification of fluorescent staining of clots

**Fig. 2:**
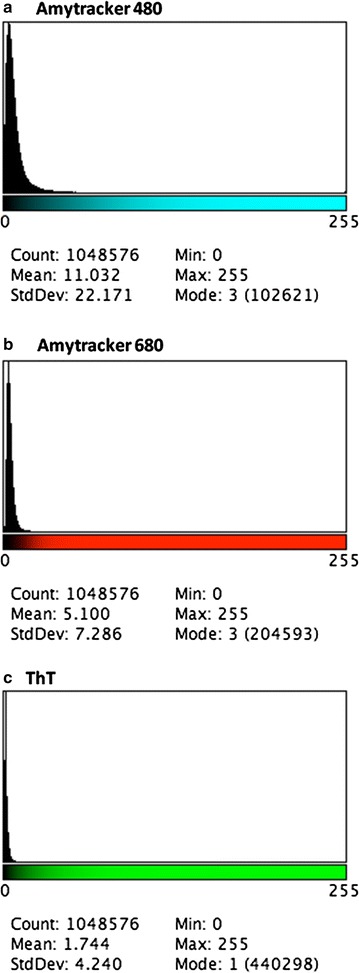
Example of the histograms generated from diabetes clots with added fluorescent markers. (The x-axis represents the grey value range (8-bit greyscale [0, 255]) and the y-axis indicates the number of total pixels for each grey value.) The histograms were generated using FIJI and every composite confocal micrograph contains data of all 3 fluorescent markers. In FIJI the channels can be split and analysed separately, using the histogram function. **a** Amytracker™ 480; **b** Amytracker™ 680; **c** ThT

We assessed the variance between (black) background and the presence of fluorescent pixels (binary comparison) for each of the three fluorescent markers in the clots. See [10, 11] for a detailed explanation of the methods. We used the histogram function in ImageJ (FIJI) and calculated the coefficient of variation (CV) (as SD/mean) of the histogram of different pixel intensities as our metric to quantify and discriminate between clots of healthy (age-controlled) naïve PPP and clots from T2D individuals. Figure 2 gives an example of the 3 marker histograms (Amytracker™ 480, 680 and ThT). CVs were calculated from the data shown at the bottom of each histogram.

### Structured illumination super-resolution (SR-SIM) microscopy

We also prepared structured illumination super-resolution (SR-SIM) microscopy [60] Z-stacks of T2D clots and compared the T2D clots to clots where we previously added known amyloidogenic molecules (iron, LPS, LTA1 and LTA2) to PPP from healthy donors (unpublished data from [11]). Previously, we incubated the four candidate amyloidogenic molecules for 1 h before adding the three fluorescent markers.

### Statistical analysis

Sample analysis was performed by the Mann–Whitney U test, using the STATSDIRECT (version 2.8.0) software and with GraphPad Prism (version 5.0) and one-way ANOVA with the Kruskal–Wallis non-parametric test and the Dunns post-test.

## Results

**Fig. 3:**
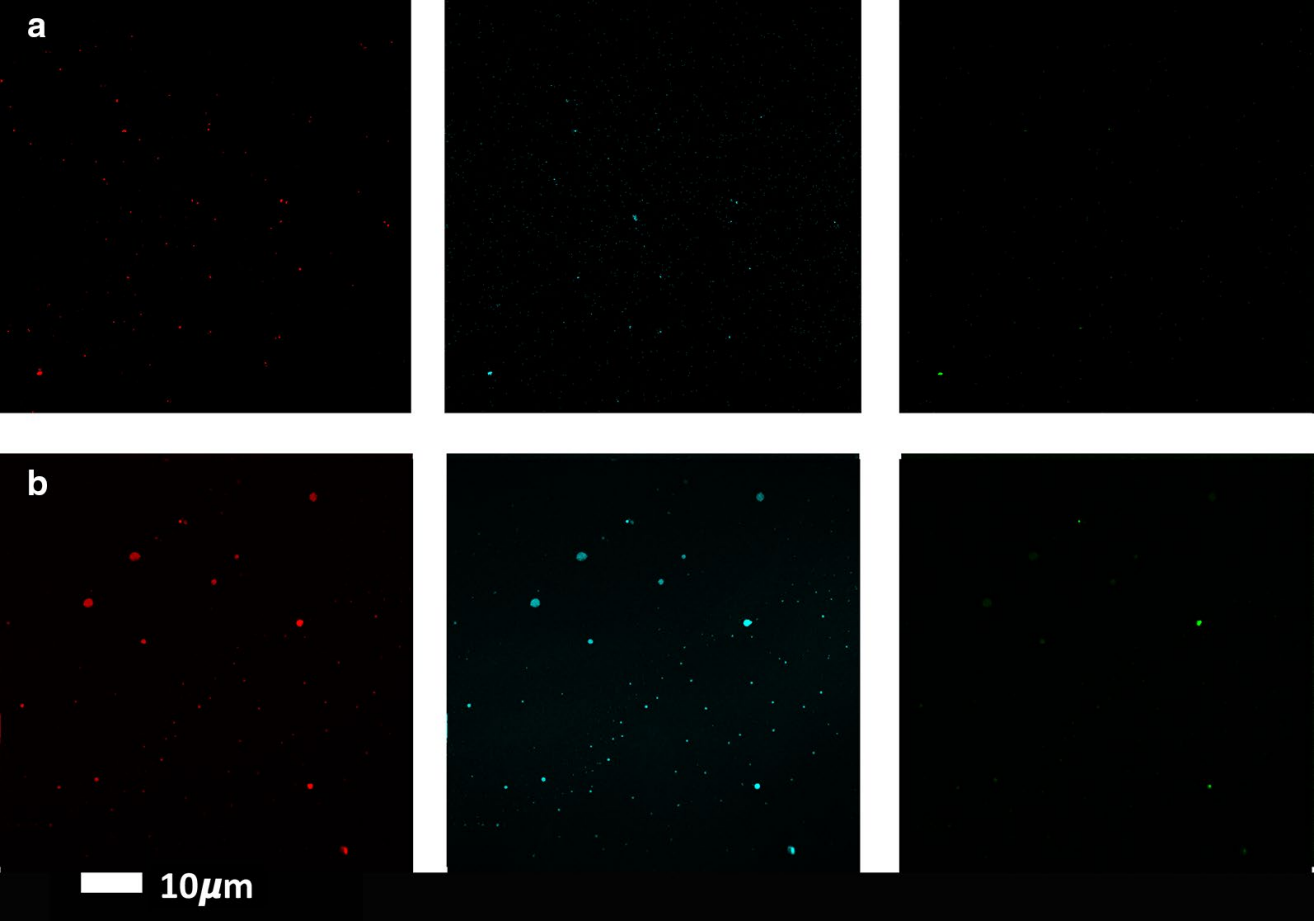
Clots from representative healthy individuals with added amyloid stains. **a** young individual (median: 22 years) and **b** age-controlled individual (median: 61 years) and from left to right Amytracker 680 (red), Amytracker 480 (blue) and ThT (green)

**Fig. 4:**
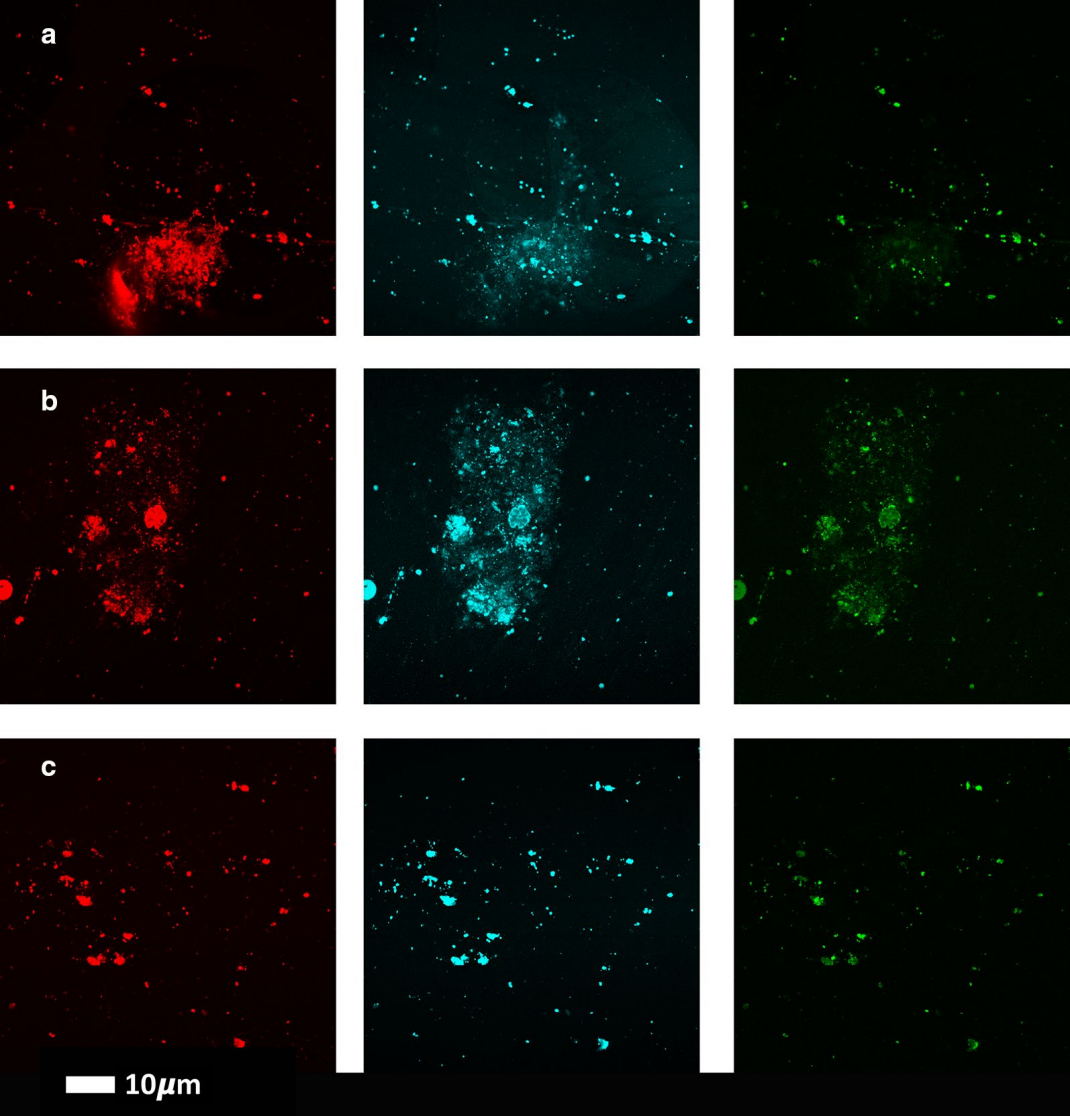
Clots from three type 2 diabetes individuals with added amyloid–specific fluorescent markers (rows **a** to **c** represent 3 different individuals with type 2 diabetes); columns from left to right Amytracker 680 (red), Amytracker 480 (blue) and ThT (green)

**Fig. 5:**
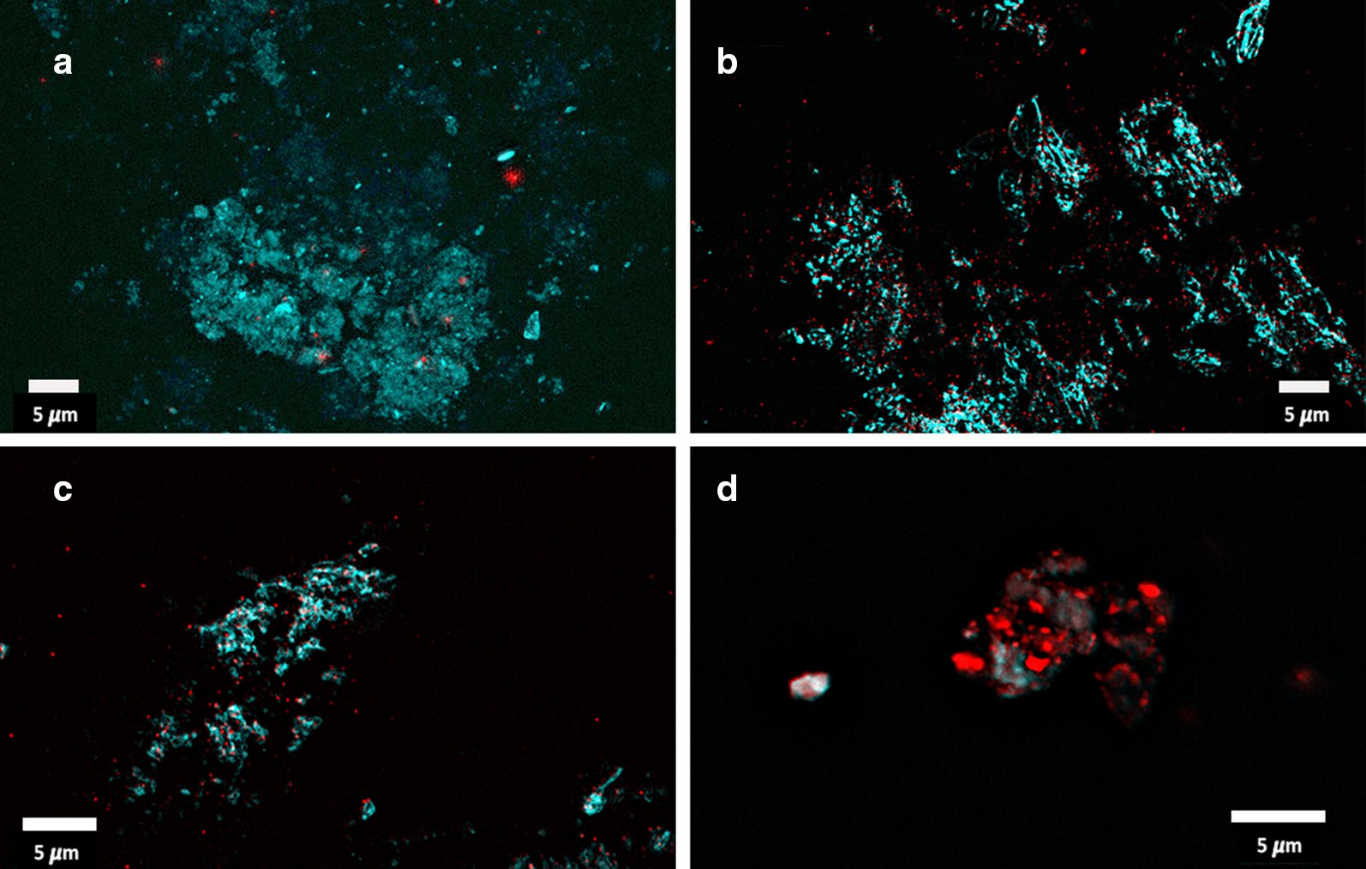
Z-stacks from **a** type 2 diabetes, **b** healthy clot with added LPS (*E. coli*), **c** healthy clot with added LTA1 (*S. aureus*), and **d** healthy clot with added LTA2 (*S. pyogenes*)

Table 1 shows demographics of our control groups and T2D sample, while Table 2 shows CV results which are calculated by using the mean and SD of the fluorescence for each of the three fluorescent markers in the different clots (Materials and Methods). There were no significant differences in CVs for the three markers when we compared the young and the old control PPP clot structure, and we therefore conclude age does not appear to influence our experimental design. However, there were significant differences in CVs for all three markers when the healthy (age-matched) individuals and T2D clots were compared. Figures 3 and 4 show confocal microscopy of PPP for representative clots from healthy and T2D samples. Figure 5 shows SR-SIM z-stacks of a representative T2D clot and also that of a healthy individual with added LPS, LTA1 and LTA2 (unpublished data from [11]). In addition, we did not find significant differences between our normal and overweight BMI control groups, therefore in this paper, we group both the normal BMI and overweight individuals as the control sample for the T2D sample.

**Table 1:** Demographic data of controls and type 2 diabetes

Healthy individuals (N = 34)							
	Gender			Age			BMI: normal: < 24.9; overweight: > 25
%; median; STD	Age-controlled healthy individuals (n = 17)						
	F: 82%; M: 18%			61 (± 11)			< 24.9: 47%; > 25: 53%
	Young healthy individuals (n = 17)						
	F: 24% M: 76%			22 (± 4)			< 24.9: 88%; > 25: 12%

Type 2 diabetes individuals (N = 33; BMI: > 25)							
	Gender	Age	HbA1c (%)	Chol (mMol L^−1^)	% with dyslipidaemia (%)	% with hypertension (%)	% with using anti-coagulants (%)
%; median; STD	F: 39% M: 61%	62 (± 11)	7 (± 1.2)	3.95 (± 0.74)	79	61	55

Statistical analysis was performed with the Mann–Whitney U test, using the STATSDIRECT (version 2.8.0) software

**Table 2:** Results from coefficient of variation (CV calculated from confocal micrographs of clots from healthy and diabetes plasma)

Confocal data analysis				
Confocal data: young (n = 17) versus old (= 17) healthy individuals				
	Marker	Healthy clot CV data (age-controlled for T2D)	Healthy young clot CV data	P
%; median; STD	ThT	1.30 (± 0.61)	1.42 (± 0.64)	P = 0.21
	Amytracker 480	0.89 (± 0.48)	0.78 (± 1.04)	P = 0.08
	Amytracker 680	1.30 (± 0.46)	1.34 (± 0.77)	P = 0.94

Confocal data: Age-controlled healthy individuals (n = 17) versus type 2 diabetes individuals (N = 33)				
	Marker	Healthy clot CV data (age-controlled)	Type 2 diabetes clot CV data	P
Median; STD	ThT	1.30 (± 0.61)	2.52 (± 1.16)	P < 0.0001
	Amytracker 480	0.89 (± 0.48)	1.44 (± 0.50)	P < 0.0001
	Amytracker 680	1.30 (± 0.46)	2.30 (± 0.67)	P < 0.0001

Confocal data: Treated versus untreated diabetes data (n = 15)				
	Marker	Untreated T2D	T2D plasma treated with 8 ng L^−1^ LBP and 250 µM ergothioneine	P
Median; STD	ThT	2.23 (± 1.21)	1.58 (± 0.78)	P = 0.0008
	Amytracker 480	1.25 (± 0.42)	1.25 (± 0.54)	P = 0.37
	Amytracker 680	1.91 (± 0.73)	1.37 (± 0.55)	P = 0.0009

	Marker	Untreated T2D	T2D plasma treated with 20 ng L^−1^ LBP and 250 µM ergothioneine	P
Median; STD	ThT	2.23 (± 1.21)	1.75 (± 0.61)	P = 0.007
	Amytracker 480	1.25 (± 0.42)	0.90 (± 0.34)	P = 0.0001
	Amytracker 680	1.91 (± 0.73)	1.50 (± 0.37)	P = 0.0002

	Marker	Untreated T2D	T2D plasma treated with 30 ng L^−1^ LBP	P
Median; STD	ThT	2.23 (± 1.21)	1.87 (± 0.60)	P = 0.033
	Amytracker 480	1.25 (± 0.42)	1.45 (± 2.98)	P = 0.036
	Amytracker 680	1.91 (± 0.73)	1.70 (± 0.60)	P = 0.042

Statistical analysis was performed with the Mann–Whitney U test, using the STATSDIRECT (version 2.8.0) software

The clots from the T2D patients most resemble that of the controls incubated with LPS. We have previously suggested that the potent inflammagens LPS and LTA can cause aberrant clot formation where the fibrin(ogen) protein becomes amyloid(ogenic) after exposure to these inflammagens [11]. In this paper, our results show that in T2D, amyloidogenesis is also present, as confirmed by the 2 LCO markers, and are similar to clots from healthy individuals but where LPS and LTA had been added prior to coagulation. We thus suggest that this aberrant morphology may (at least in part), be due to the presence of bacterial inflammagens in T2D blood.

**Fig. 6:**
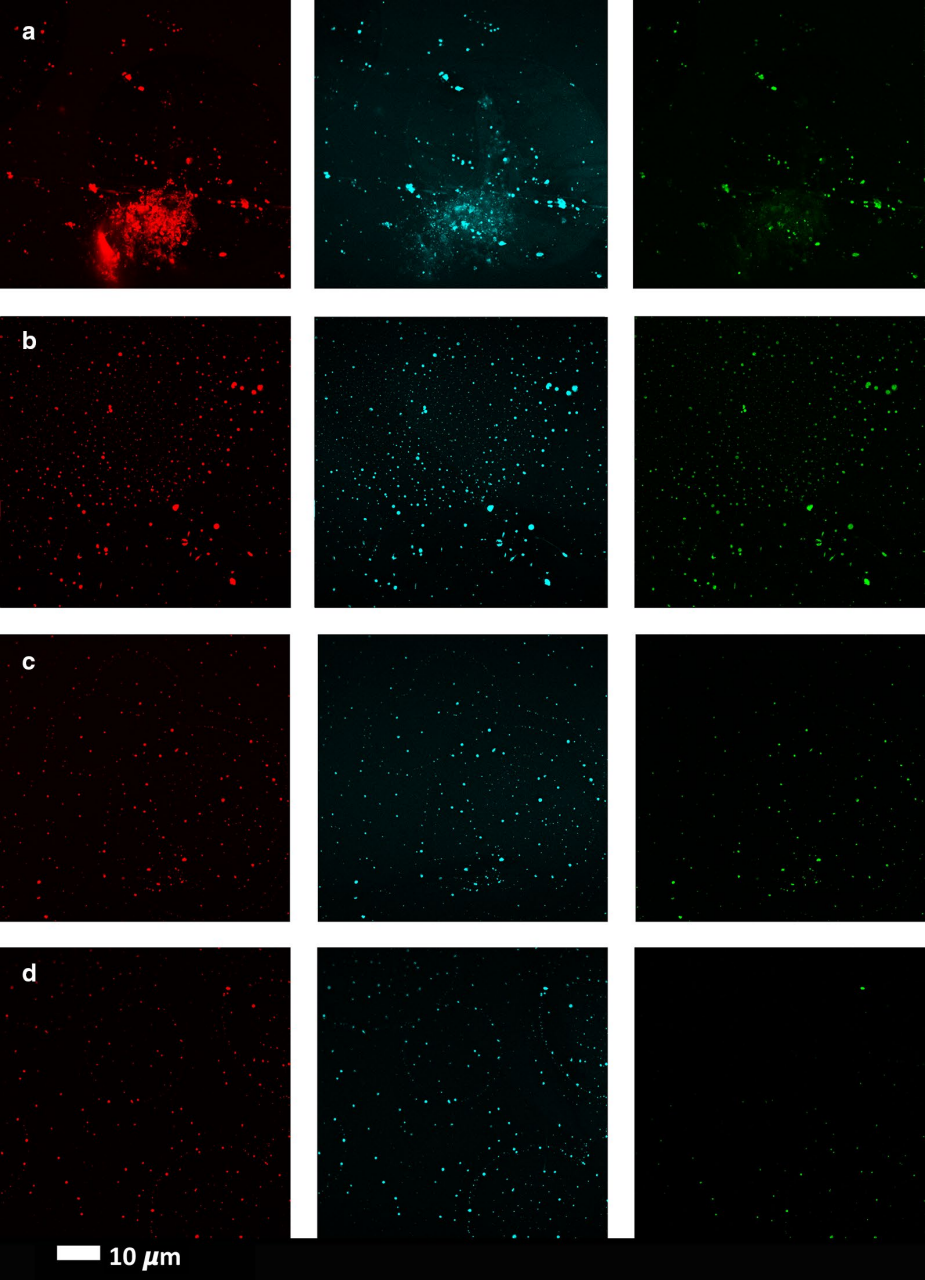
Clots from a representative type 2 diabetes individual with added LPS-binding protein (LBP) and ergothioneine, and fluorescent markers. **a** Naïve T2D clot; **b** 8 ng L^−1^ LBP and 250 µM ergothioneine; **c** 20 ng L^−1^ LBP and 250 µM ergothioneine **d** 30 ng L^−1^ LBP. Fluorescence shown from left to right Amytracker 680 (red), Amytracker 480 (blue) and ThT (green)

**Fig. 7:**
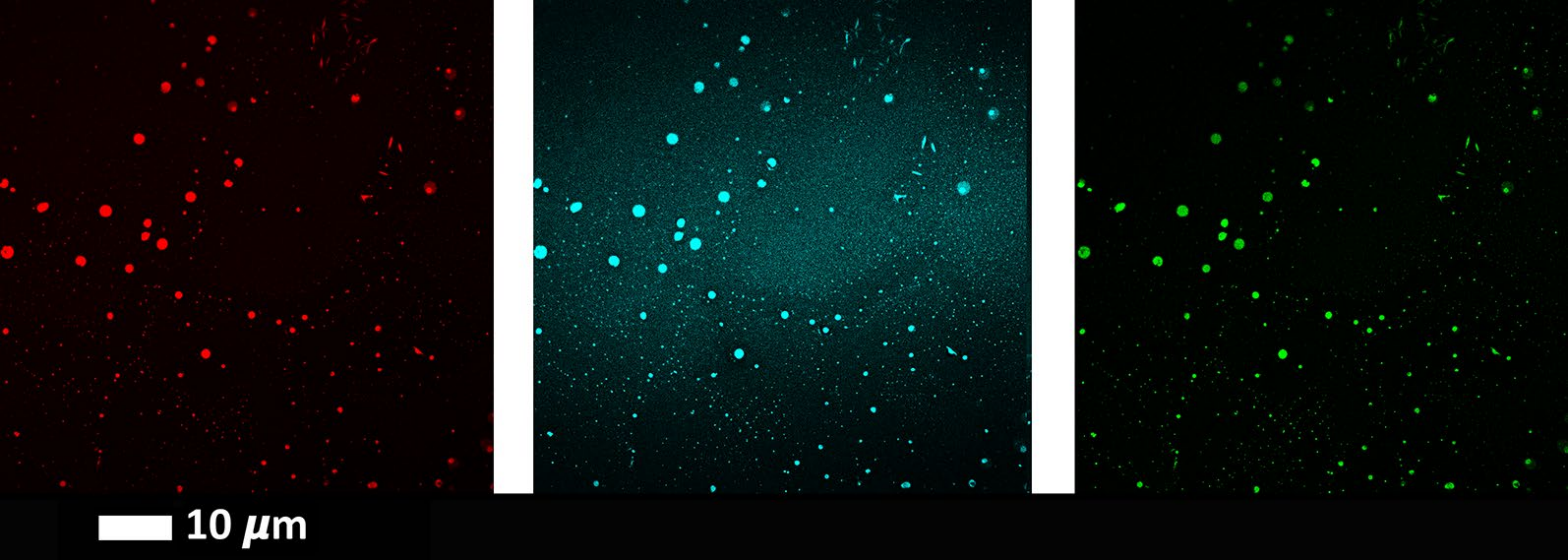
T2D with added 250 µM ergothioneine. Fluorescence shown from left to right Amytracker 680 (red), Amytracker 480 (blue) and ThT (green)

**Fig. 8:**
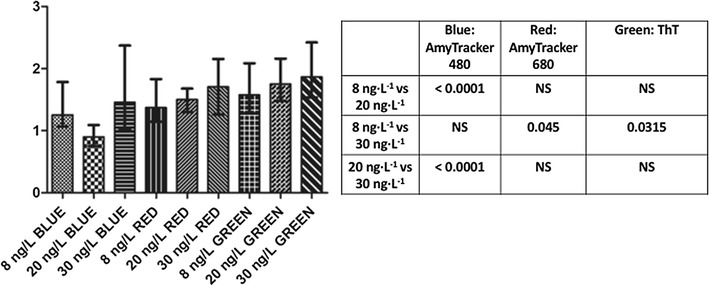
Graph drawn from the confocal results [(CVs) of the various LBP exposures to type 2 diabetes (8, 20 and 30 ng L^−1^ LBP]. Sample analysis was performed with GraphPad Prism (version 5.0) and one-way ANOVA with the Kruskal–Wallis non-parametric test and the Dunns post-test

We also added various concentrations of LPS-binding protein (LBP) and the antioxidant ergothioneine to T2D plasma to determine if the amyloid signal from the LCO dyes will be reduced. See Table 2 for the results as well as Fig. 6 for confocal data. These results show a dose-response in the reduction of fluorescent signal as seen with CV data. LBP breaks up the amyloid signal, where fewer large fluorescent amyloid clumps are noted, but rather more small fluorescent spots are seen (see Fig. 6). These data also show rather clearly the differences of detail between the binding (sites) of the three fluorophores. Ergothioneine does not significantly reduce the amyloid by itself; however, the effects of the addition of other antioxidants needs to be explored further (Fig. 7). Figure 8 shows a comparison of the confocal results [(CVs) of the various LBP exposures to T2D plasma (8, 20 and 30 ng L^−1^ LBP], using a one-way ANOVA with the Kruskal–Wallis non-parametric test and the Dunns post-test.

**Fig. 9:**
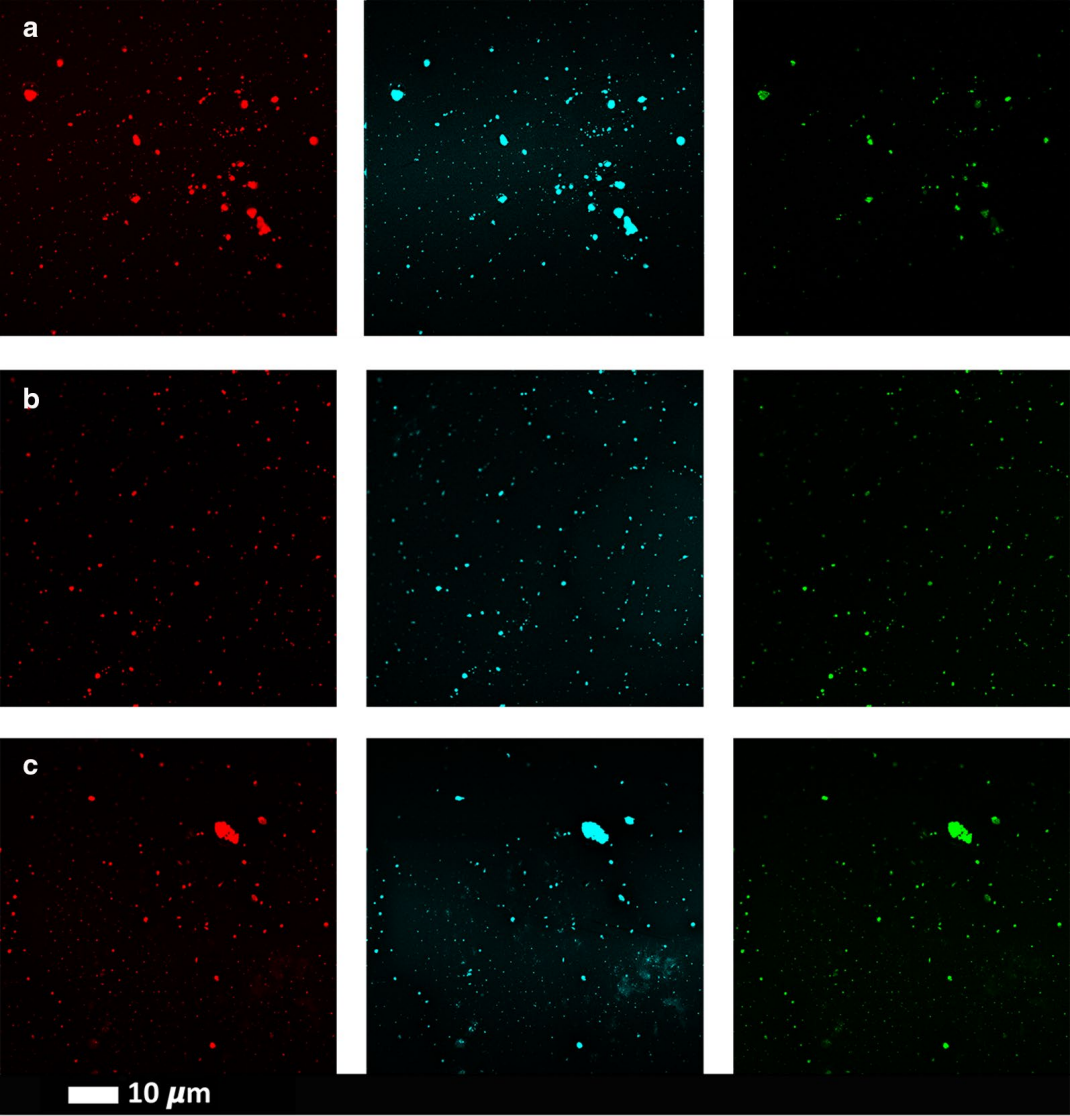
**a** Purified fibrinogen with 0.4 ng L^−1^ LPS and added thrombin **b** Purified fibrinogen with 0.4 ng L^−1^ LPS followed by 30 ng L^−1^ LBP and thrombin **c** Purified fibrinogen with 0.4 ng L^−1^ LPS and 250 µM ergothioneine followed by thrombin. Fluorescence shown from left to right Amytracker 680 (red), Amytracker 480 (blue) and ThT (green)

Purified fibrinogen with added 0.4 ng L^−1^ LPS followed by increasing concentrations of LBP with and without ergothioneine showed the same trends, where the amyloid signal was dispersed and appeared as small fluorescent spots (see Fig. 9).

## Discussion

T2D is one of many chronic, inflammatory diseases, and as such, shares a variety of hallmarks with these other conditions. These include the presence of inflammatory cytokines, iron dysregulation, and various coagulopathies [61–65]. What has not been clear is the actual cause of this inflammation; that is, what are the stimulating molecules to which inflammation is a response? Such a cause must exist, as there is otherwise little reason why the inflammation might happen ‘spontaneously’ in a way that it does not in healthy individuals. A strong candidate cause of such systemic inflammation may lie in a dormant microbiome that can shed inflammagens. In nature, most microbes exist in a dormant, non-replicating state, and as such are typically difficult to culture using standard microbiological culture techniques (e.g. [66, 67]). A lack of culturability may mean that a cell is non-viable under the circumstances tested, but they might be culturable in that they may be induced to return to a state of culturability (by a process or processes typically referred to as ‘resuscitation’) [66, 67].

We have suggested [68–71] that the source of these inflammagens is in fact populations of dormant microbes that are resident in blood and tissues, and that can occasionally ‘wake up’, often in response to bioavailable iron, whereupon they shed known, potent inflammagens such as LPS and LTA [11]. LPS has also been specifically implicated in T2D pathology [18–25, 72]. It is suggested that LPS may contribute to low-grade systemic inflammation in insulin-resistant states, and it is also now accepted that specifically gut bacteria is the sources of LPS [24]. In T2D it is well-known that there is an increased intestinal permeability in the genesis of T2D [73–75], and that this can be the origin for LPS.

We have also shown, using scanning electron microscopy (e.g. [1, 8, 69, 76–78], that in many cases a particular manifestation of the coagulopathies accompanying these diseases is the clotting of blood into a highly anomalous form. The above two strands of work led to the idea that the anomalous clotting might in fact be caused by the presence of low concentrations of bacterial–derived LPS, and this turned out to be the case when we tested the addition of extremely low concentrations of LPS to PPP from healthy individuals [9]. Further [9, 77], the fact that these clots could be stained with the amyloid stain thioflavin T, and blocked by the addition of LBP, strongly suggested (i) that the anomalous clotting was amyloid in nature, and (ii) that LPS (and the potent inflammagen LTA from Gram-positive bacteria) could indeed be a typical culprit. We recently showed that both LPS and LTA and also iron, cause healthy fibrin(ogen) to become amyloidogenic, and we used Amytracker™ 480, 680 and ThT in these experiments [11]. We found that the nature of the staining efficiencies also varied depending on the inflammagen added, with LTA particularly leading to preferential staining by Amytracker™ 680.

A logical corollary of the above, then, was that the anomalous clotting seen in PPP from patients with chronic inflammatory diseases might also be due (at least in part) to the presence of LPS, and that this too might be reversed by the addition of LBP. We had illustrated this previously using the common stain thioflavin T [59, 77] and here show it further using a variety of microscopic techniques, together with two novel stains, viz. Amytracker™ 480 and Amytracker™ 680. In all cases, there was a very substantial staining of the PPP from T2D patients, and this was removed, in a dose-dependent fashion, by the pre-incubation of the PPP with relatively low concentrations of lipopolysaccharide binding protein. In addition, the detailed nature of the staining varied for the three stains, suggested that they had both common and separate binding sites, with the two Amytracker™ stains being the most potent stains.

Ergothioneine is a potent antioxidant that is resistant to autoxidation [53, 54]. However, it was without effect on the amyloid staining, suggesting that there is no redox-dependent basis for these effects. This said, the effects of the addition of other antioxidants and/or polyphenolic compound need to be explored further.

Overall, we have shown very clearly that there is a substantial potential for amyloidogenesis when the plasma of individuals with T2D is clotted, and that this can be prevented by preincubation of the PPP with lipopolysaccharide-binding protein. This suggests very strongly that there is indeed a microbial component involved in the development of T2D and its sequelae. Of course there are potentially other circulating inflammatory molecules in T2D blood that might contribute to the amyloidogenic fibrin(ogen) structure, and some may include iron, LTA, SAA and upregulated cytokines. However, in this paper, we show that LPS/LTA is one of the important inflammagens in T2D, and the LBP removes most of the molecules that causes the amyloid formation in fibrin(ogen). Considering that amyloids can be cytotoxic, and many of the sequelae of chronic T2D involve damage to cells of other tissues such as the kidney (nephropathies) and the eye (retinopathies), it is at least reasonable that treatments designed either to remove the dormant microbes or the use of LBP to remove their cell wall products might be of therapeutic benefit. This is an important question for the future.
